# Evaluation of *Lycium chinense* Germplasms in China Based on Fruit Quality Traits

**DOI:** 10.3390/plants15101506

**Published:** 2026-05-15

**Authors:** Zijing Guo, Chaoguang Yu, Yan Lu, Wanwen Yu

**Affiliations:** 1 National Key Laboratory for Development and Utilization of Forest Food Resources, Co-Innovation Center for Sustainable Forestry in Southern China, Nanjing Forestry University, Nanjing 210037, China; 18506335567@163.com; 2 College of Forestry, Henan Agricultural University, Zhengzhou 450046, China; 3 Jiangsu Key Laboratory for Conservation and Utilization of Plant Resources, Institute of Botany, Jiangsu Province and Chinese Academy of Sciences (Nanjing Botanical Garden Mem. Sun Yat-Sen), Nanjing 210014, China; yuchaoguang168@cnbg.net

**Keywords:** antioxidant capacity, amino acids, comprehensive evaluation, fruit, total phenolics, wolfberry

## Abstract

The fruits of *Lycium chinense* are important medicinal and edible resources with multiple bioactive functions, including hepatoprotective, antioxidant, and immunomodulatory effects. Although this species is widely distributed in China and exhibits abundant germplasm resources, systematic evaluations of fruit quality variation among wild germplasm remain limited, restricting the selection and breeding of superior resources. In this study, eight wild germplasm resources of *L. chinense* were collected from the eastern coastal regions of China, including Liaocheng (LC), Rugao (RG), Dafeng (DF), Suzhou (SZ), Qidong (QD), Dongtai (DT), Jingjiang (JJ), and Sheyang (SY). A total of 29 fruit quality-related traits, including fruit size, flavonoids, soluble sugars, vitamin C, and amino acids, were analyzed. Significant differences were observed among germplasm resources in both fruit size and internal quality. Comprehensive evaluation based on principal component analysis and entropy weight–grey relational analysis indicated that SY and LC ranked highest. SY exhibited smaller fruits but superior nutritional quality, with higher levels of soluble protein, vitamins, and amino acids. In contrast, LC showed larger fruits and higher contents of polysaccharides and total phenolics, along with stronger antioxidant capacity. Overall, SY and LC represent promising germplasm resources for breeding and utilization of *L. chinense*.

## 1. Introduction

Wolfberry, commonly referred to as *L. barbarum* or *L. chinense*, belongs to the Solanaceae family and is widely used as traditional Chinese medicinal materials and functional foods [1,2]. Wolfberry fruits are rich in phytochemicals, including polysaccharides, phenolic compounds, carotenoids and vitamins [3]. These bioactive compounds are associated with various health-promoting effects, such as antioxidant, anti-inflammatory, hypoglycemic, and hepatoprotective activities [3]. Owing to these nutritional and pharmacological properties, wolfberry has attracted increasing attention from the food, pharmaceutical, and nutraceutical industries. To date, *L. barbarum* is widely cultivated in northwestern China, particularly in Ningxia, and is valued for its favorable fruit quality, high polysaccharide content, and strong antioxidant activity [4]. In addition, *L. barbarum* fruits are extensively exported to numerous countries and regions, including North America, Europe, and Australia [5]. As a functional food, it fulfills a growing global market demand driven by the increasing recognition of its health-promoting properties [6]. Consequently, the annual production value of the *L. barbarum* industry has reached approximately 21 billion RMB, highlighting its significant economic importance [7].

In contrast to *L. barbarum*, *L. chinense* belongs to the Solanaceae family and is native to or cultivated in East Asia, including China, Korea, and Japan [8]. It is less commercially cultivated, but is widely distributed in eastern, central, and southern China, where abundant natural germplasm resources are present [9]. EST-SSR studies have revealed that natural germplasm resources of *L. chinense* have genetic variation shaped by geography and hybridization, which may underlie phenotypic differences [10]. Additionally, *L. chinense* fruit extracts showed hepatoprotective effects in vitro [11] and in vivo by alleviating chemical-induced liver injury through antioxidant and anti-inflammatory mechanisms [12]. In addition, *L. chinense* fruits have been reported to exhibit antioxidant, immunomodulatory, and anti-aging activities, and may contribute to the prevention of Alzheimer’s disease and related neurodegenerative disorders [2,13]. This broad geographic distribution, rich genetic diversity and notable pharmacological efficacy underscore its potential value for further development.

Further development of *L. chinense* requires genetic improvement, which is essential for the medicinal and edible plant industry and depends on the evaluation and utilization of germplasm resources. Systematic germplasm evaluation provides a foundation for identifying elite accessions with desirable agronomic traits, nutritional quality, and bioactive compound profiles, thereby supporting targeted breeding programs. For instance, evaluations of morphological traits, yield and key bioactive components have been conducted in various medicinal and edible plants, including *Salvia miltiorrhiza* and *Panax ginseng*, leading to the selection and release of improved cultivars [14,15,16]. Similar studies have also been conducted in *L. barbarum* [17,18]. Polysaccharide yield, structural features and monosaccharide composition, as well as flavonoids composition differed markedly among *L. barbarum* fruits from Ningxia, Qinghai, and Gansu [17,19]. Based on these germplasm evaluation studies, several elite cultivars, such as Ningqi series, have been successfully developed [20]. Although possessing broad geographic distribution and abundant genetic diversity, *L. chinense* remains largely unexplored in terms of systematic germplasm evaluation.

The eastern coastal regions of China harbor extensive wild *L. chinense* germplasm resources. The region’s relatively high temperatures, abundant sunlight, and sufficient rainfall are conducive to plant growth and fruit development, likely contributing to higher yield and superior fruit quality [21]. In addition, these fruits have long been harvested and consumed by local residents, further indicating their potential value as edible germplasm. Therefore, eight *L. chinense* germplasms collected from this region were selected for systematic evaluation. Fruit quality was assessed using multiple indicators, including morphological traits, nutritional composition and antioxidant-related metabolites. Principal component analysis (PCA) and entropy weight–grey relational analysis (EW-GRA) were used to comprehensively evaluate and rank the accessions. This study aimed to systematically evaluate the fruit quality of *L. chinense* germplasms and to explore potential differences in their nutritional and functional components. In particular, this study sought to determine whether significant variations exist among different germplasms in terms of fruit quality and bioactive compounds. These findings provide a scientific basis for germplasm utilization and the breeding of high-quality cultivars.

## 2. Results

### 2.1. Morphological Traits

**Figure 1:**
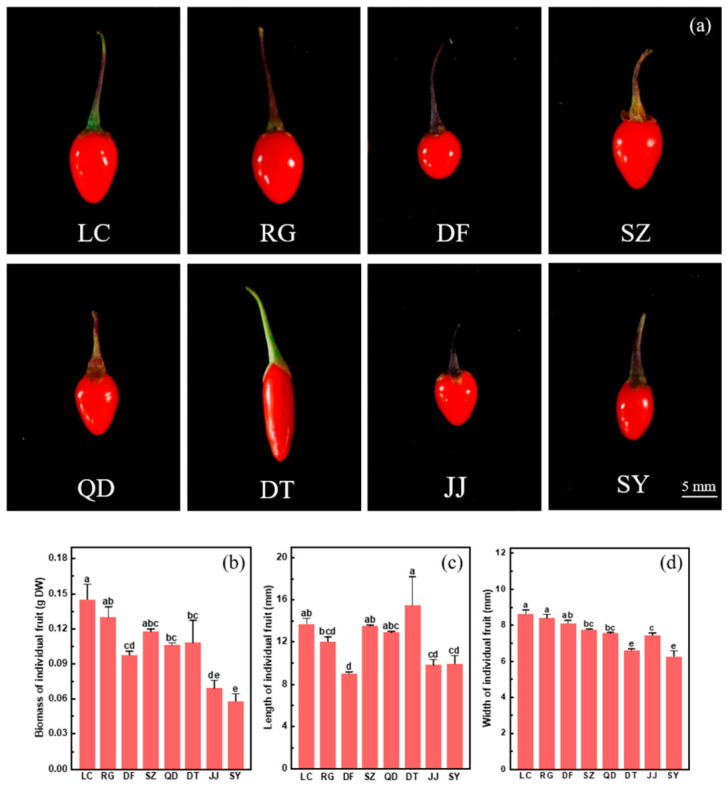
Fruit phenotypes (**a**), and biomass (**b**), length (**c**) and width of individual fruit (**d**) in eight germplasms (LC, RG, DF, SZ, QD, DT, JJ and SY) of *L. chinense*. Data indicate means ± SE (*n* = 3). Different letters on the bars indicate significant differences between the germplasms.

The shape, biomass, length and width of fruit varied greatly among the eight germplasms of *L. chinense* (Figure 1). It was obvious that the fruit shapes of these germplasms are classified into four types: elliptic (RG and SY), spherical (DF), elongated elliptic (DT) and ovoid (LC, SZ, QD and JJ) (Figure 1a). Notably, the fruit shape of DT was similar to that of *L. barbarum* (Figure 1a). Among the germplasm resources, fruit biomass varied from 0.06 to 0.14 g, fruit length from 9.02 to 15.46 mm, and fruit width from 6.24 to 8.61 mm (Figure 1b–d). Generally, LC and RG ranked among the highest in fruit biomass, length, and width, whereas JJ and SY ranked among the lowest for these traits (Figure 1b–d). SZ and QT showed intermediate values for most measurements (Figure 1b–d).

### 2.2. Phenolics, Flavonoids, Polysaccharidss and Antioxidant Capacity

**Figure 2:**
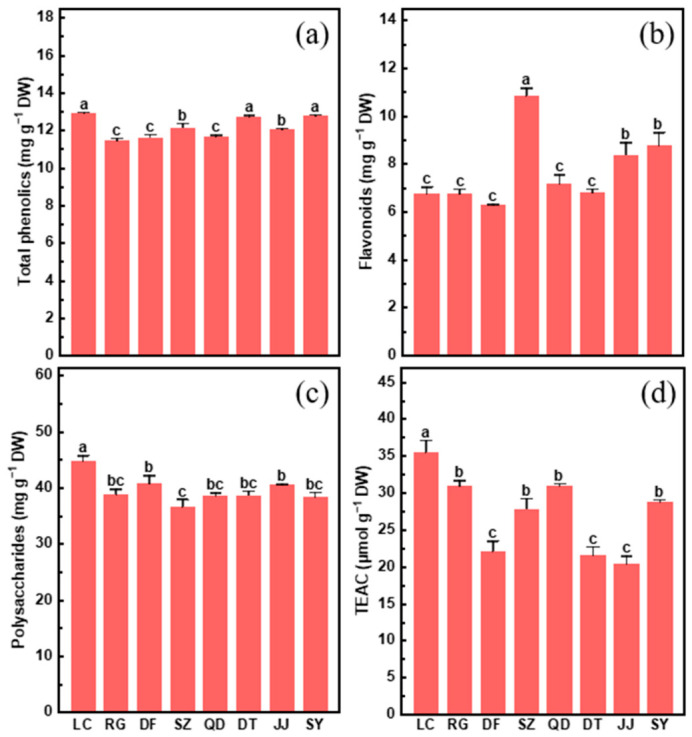
Concentrations of total phenolics (**a**), flavonoids (**b**), polysaccharides (**c**) and TEAC (**d**) in the fruits of eight *L. chinense* germplasms (LC, RG, DF, SZ, QD, DT, JJ and SY). Data indicate means ± SE (*n* = 3). Different letters on the bars indicate significant differences between the germplasms.

Phenolic compounds, flavonoids, and polysaccharides are important bioactive compounds that contribute to antioxidant capacity and human health [22,23]. The concentrations of phenolics, flavonoids and polysaccharides varied among the eight germplasms of *L. chinense* (Figure 2a–c). Specifically, polysaccharides and flavonoids varied more markedly, while total phenolics remained relatively stable across germplasms (Figure 2a–c). Total phenolics ranged from 11.47 to 12.91 mg g^−1^, with LC, DT and SY exhibiting the highest level and RG, DF and QD the lowest (Figure 2a). Flavonoids content varied from 6.30 to 10.85 mg g^−1^, with SZ showing the greatest level and DF, RG, LC, QD and DT the lowest (Figure 2b). Polysaccharides level ranged from 36.64 to 44.75 mg g^−1^, with LC exhibiting the highest level and SZ the lowest (Figure 2c). Trolox equivalent antioxidant capacity (TEAC) value, representing antioxidant capacity, varied from 22.07 to 27.87 mg g^−1^, with LC showing the highest level and DF, DT and JJ the lowest (Figure 2d).

### 2.3. Nutritional Composition

**Figure 3:**
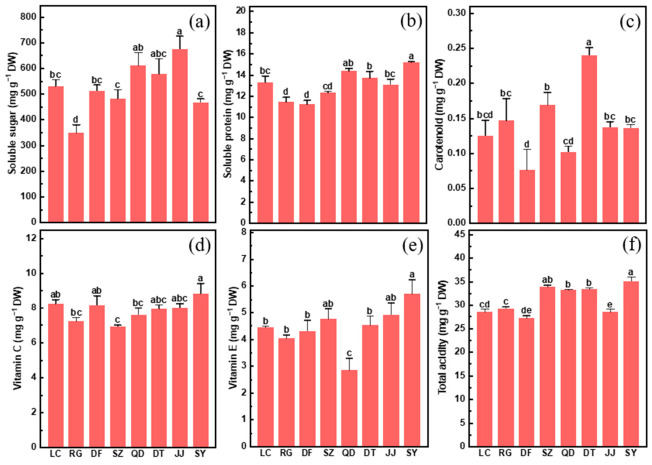
Concentrations of soluble sugar (**a**), soluble protein (**b**), carotenoid (**c**), vitamin C (**d**), vitamin E (**e**) and total acidity (**f**) in the fruits of eight *L. chinense* germplasms (LC, RG, DF, SZ, QD, DT, JJ, and SY). Data indicate means ± SE (*n* = 3). Different letters on the bars indicate significant differences between the germplasms.

In fruit quality evaluation, sensory attributes and nutritional composition are considered important indicators of utilization potential [24,25]. Nutritional composition varied among the eight wild *L. chinense* germplasm resources (Figure 3). Specifically, soluble sugar and carotenoid contents showed greater variation, while total acidity and soluble protein remained relatively stable across germplasms (Figure 3). Soluble sugar content varied from 296.93 to 756.12 mg g^−1^, with JJ showing the highest level and RG the lowest (Figure 3a). Carotenoid content ranged from 0.037 to 0.261 mg g^−1^, with DT showing the highest level and DF the lowest (Figure 3c). Among the concentrations of soluble protein (10.53–15.36 mg g^−1^), vitamin C (6.84–9.71 mg g^−1^), and vitamin E (2.38–6.59 mg g^−1^), along with total acidity (4.82–6.68 mg g^−1^), SY revealed the highest level, while the lowest values were observed in DF and RG (protein), SZ (vitamin C), QD (vitamin E), and JJ (acidity), respectively (Figure 3b,d–f).

**Figure 4:**
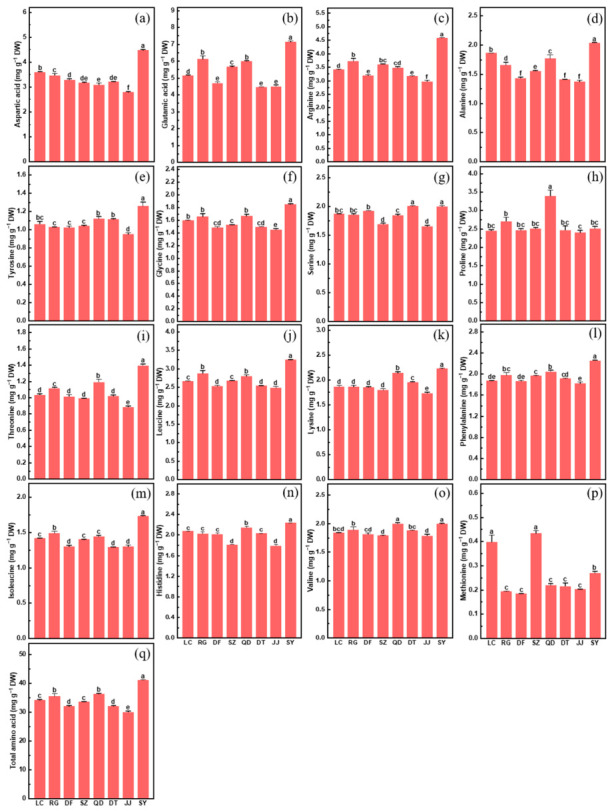
Concentrations of aspartic acid (**a**), glutamic acid (**b**), arginine (**c**), alanine (**d**), tyrosine (**e**), glycine (**f**), serine (**g**), proline (**h**), threonine (**i**), leucine (**j**), lysine (**k**), phenylalanine (**l**), isoleucine (**m**), histidine (**n**), valine (**o**), methionine (**p**) and Total amino acid (**q**) in the fruits of eight *L. chinense* germplasms (LC, RG, DF, SZ, QD, DT, JJ and SY). Data indicate means ± SE (*n* = 3). Different letters on the bars indicate significant differences between the germplasms.

Amino acids, as fundamental components of proteins, play important roles in plant growth, and fruit nutritional quality [26]. The concentrations of amino acids varied markedly among the *L. chinense* germplasms (Figure 4). Most amino acids exhibited similar variation patterns, with highest levels in SY and lowest levels in JJ. Among non-essential amino acids, aspartic acid, glutamic acid, and arginine were the most abundant, ranging from 2.79 to 4.47 mg g^−1^, 4.47 to 7.15 mg g^−1^, and 2.98 to 4.57 mg g^−1^, respectively (Figure 4a–c). Other non-essential amino acids, including alanine, tyrosine, glycine, and serine, exhibited comparatively narrower variation (Figure 4d–g). Proline showed a distinct variation pattern, ranging from 2.39 to 3.39 mg g^−1^, with QD exhibiting the highest level and JJ the lowest (Figure 4h). For essential amino acids, threonine, leucine, lysine, phenylalanine, isoleucine, histidine, and valine showed similar variation trends, with highest levels in SY and lowest levels in JJ (Figure 4i–o). Methionine had the highest level in SZ, followed by LC, and the lowest in DF (Figure 4p). Total amino acid content ranged from 30.08 to 41.19 mg g^−1^ (Figure 4q), with SY showing the highest level and JJ the lowest.

### 2.4. Correlation Analysis

**Figure 5:**
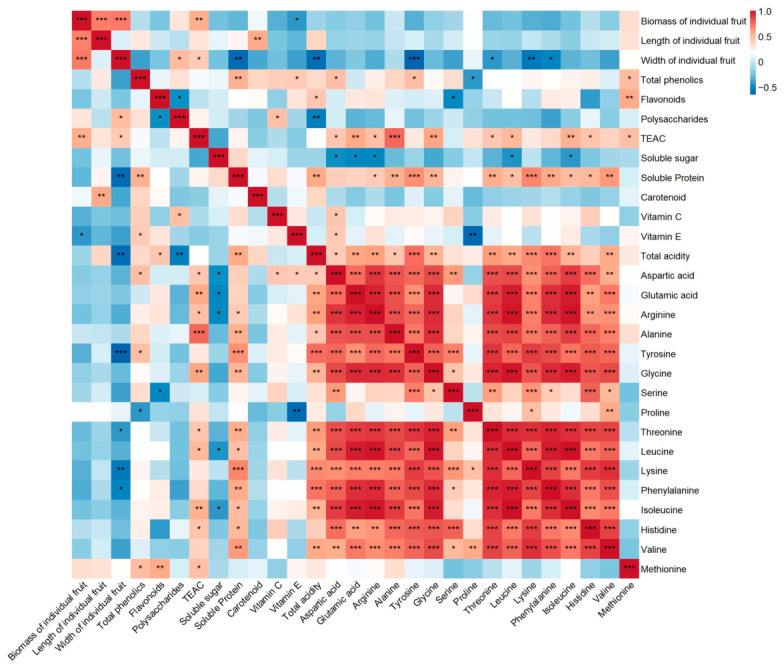
Pearson correlation analysis of 29 fruit quality traits in eight *L. chinense* germplasms. Red and blue colors represent positive and negative correlations, respectively, and color intensity reflects the strength of the correlation. Asterisks indicate statistically significant correlations (* *p *< 0.05, ** *p *< 0.01, *** *p* < 0.001).

Pearson correlation analysis of 29 phenotypic and physiological indicators in *L. chinense* identified 406 pairs of variables, among which 284 pairs showed highly significant correlations (*p* < 0.01) and 347 pairs revealed significant correlations (*p* < 0.05) (Figure 5), indicating extensive interactions among fruit quality traits. Fruit morphological traits were significantly associated with metabolic indicators, with fruit biomass, length, and width showing significant correlations with several amino acids and functional components (Figure 5), suggesting a linkage between fruit size and the accumulation of nutrients and bioactive compounds.

For functional components, TEAC exhibited significant positive correlations with several amino acids, including alanine, glutamic acid, leucine, and glycine (Figure 5), indicating a potential association between antioxidant capacity and amino acid composition. Regarding nutritional traits, soluble sugar showed a negative correlation with total acidity (Figure 5), reflecting a balance between sweetness and acidity. In addition, soluble sugar was negatively correlated with several amino acids (Figure 5), suggesting a potential trade-off between carbon- and nitrogen-related traits. Most amino acids were positively correlated with each other (Figure 5), indicating coordinated variation among amino acid components. Overall, these results demonstrate coordinated relationships among fruit morphological traits, nutritional traits and functional components, which may collectively contribute to variation in fruit quality in *L. chinense*.

### 2.5. Principal Component Analysis

Principal component analysis was conducted based on fruit morphological traits, antioxidant-related indices and nutritional components (Table 1). The first principal component explained 44.32% of the total variance, followed by the second, third, fourth and fifth principal component accounting for 12.57%, 10.56%, 9.43%, and 9.17% of the variance, respectively (Table 1). These five principal components accounted for 86.049% of the total variance (Table 1), indicating that they sufficiently described the major variation in fruit quality among different accessions.

**Figure 6:**
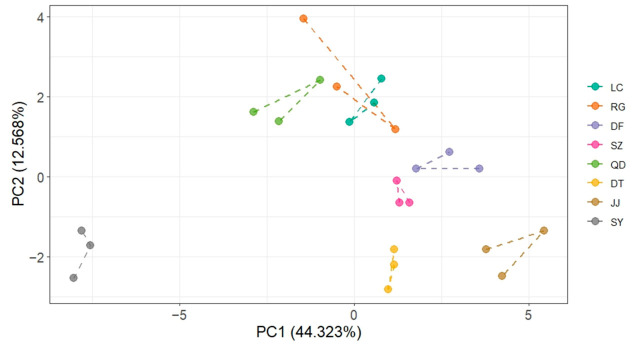
PCA plot of fruit quality traits in eight *L. chinense* germplasms.

The PCA score plot revealed a clear separation among *L. chinense* accessions (Figure 6). PC1 accounted for the largest proportion of variance and served as the primary axis separating the germplasm resources, with SY and JJ showing the greatest separation along this component (Figure 6). PC1 primarily reflected variation in nutritional traits, with several amino acids, including glycine, threonine, leucine, and phenylalanine, identified as major contributors (Table 1). PC2 was mainly associated with fruit morphological traits and antioxidant capacity, with fruit width, biomass, and TEAC contributing substantially to this component (Table 1). These results indicate that amino acid composition, fruit morphology, and antioxidant properties represent the major dimensions underlying fruit quality variation in *L. chinense*.

The 29 nutritional indicators were included in the factor analysis. All indicators were standardized prior to analysis. Factor scores for each common factor were calculated by multiplying the standardized indicator values by the corresponding factor score coefficients and summing the products [27]. Since the first five principal components were retained for comprehensive evaluation, their variance contribution rates were normalized and used as weighting factors.

The accessions were ranked in the following order: SY > LC > RG > QD > DT > SZ > DF > JJ (Table 2).

### 2.6. Entropy Weight–Grey Relational Analysis

To further validate and comprehensively assess the germplasm resources, an EW-GRA was conducted (Table 3 and Table 4). Based on the normalized data, the entropy weight method was used to determine the objective weights of each quality indicator. The results showed that among the 29 evaluated indicators, length of individual fruit, flavonoid content, glutamic acid, proline, isoleucine, and methionine were assigned relatively higher weights of 0.047, 0.071, 0.047, 0.052, 0.048, and 0.082, respectively (Table 3).

Based on the objective weights determined above, grey relational analysis was subsequently performed to comprehensively rank the germplasm resources [28,29]. After dimensionless data processing, grey relational coefficients were calculated and integrated with entropy weights to obtain EW–GRA scores. In line with the PCA results, SY achieved the highest weighted grey relational degree of 0.712, indicating superior overall performance, whereas JJ exhibited the lowest value of 0.408 (Table 4).

## 3. Discussion

Fruit quality is an important determinant of the economic and functional values of *Lycium* germplasm resources. Previous studies have mainly focused on commercially important species, such as *L. barbarum*, whose antioxidant capacity, polysaccharide bioactivity, and phenolic composition have been widely investigated [1,30]. Among various quality traits, fruit size and appearance are important agronomic characteristics closely related to yield and market value. For consumers and the food industry, comprehensive quality assessment also considers internal nutritional composition and functional properties, including antioxidant capacity and bioactive compound content. These factors jointly determine the overall market acceptance and health value of the fruit. Therefore, multi-trait evaluation frameworks integrating morphological and biochemical characteristics have been widely applied in fruit quality assessment. For example, in blueberry, fruit quality has been evaluated based on changes in phenolic composition and antioxidant activity during fruit maturation and ripening [31]. In apple and grape, variation in fruit size, color, and other traits has been analyzed in relation to physicochemical characteristics [32,33]. These studies indicate that fruit quality is a complex trait involving multiple morphological and biochemical factors. Building on this framework, this study comprehensively evaluated *L. chinense* germplasm resources, revealing variation in both morphological and biochemical traits. Specifically, fruit size traits such as fruit biomass, length, and width tended to show positive associations with certain functional attributes including antioxidant capacity, suggesting that larger fruits may exhibit relatively higher antioxidative potential. In contrast, fruit size showed negative correlations with certain nutritional components, particularly soluble proteins and some individual amino acids, suggesting that smaller fruits may tend to accumulate higher concentrations of these metabolites. In this study, a large sample size and a multi-trait evaluation system were adopted, covering morphological traits (e.g., fruit length and width), functional traits (e.g., total phenolics, flavonoids), and nutritional traits (e.g., soluble proteins, amino acids, vitamins). The analysis revealed considerable variation in multiple quality traits among different germplasms, with internal nutritional quality (e.g., amino acids, vitamins) showing higher variation than morphological traits, indicating that these nutritional components vary considerably among different germplasms. Meanwhile, a complementary relationship was observed between fruit size and nutritional components: larger-fruited accessions tended to accumulate more soluble sugars, total phenolics, and flavonoids, while smaller-fruited accessions accumulated higher levels of soluble proteins and amino acids.

Based on PCA and EW-GRA analyses, this study systematically evaluated the fruit quality of *L. chinense* germplasm resources. The two methods showed high consistency, jointly identifying SY and LC as superior germplasm resources. Notably, SY ranked the highest overall, and its superior performance is closely associated with higher levels of soluble proteins, amino acids, and vitamins, particularly vitamin C and vitamin E, indicating strong nutritional quality and functional potential. These bioactive components are closely linked to the health-promoting effects of *L. chinense* fruits, which have been reported to exhibit antioxidant, anti-aging, immunomodulatory, and neuroprotective activities [1,2]. Soluble proteins contribute to basic nutritional supply and metabolic maintenance, and vitamins C and E function as key non-enzymatic antioxidants involved in cellular redox homeostasis and protection against oxidative damage [34,35]. Amino acids are important contributors to nutritional quality, flavor formation, and metabolic regulation, and also participate in protein synthesis, nitrogen metabolism, immune regulation, and antioxidant defense [36,37,38]. Notably, under the same cultivation environment, the quality traits of all germplasms exhibited certain correlation patterns. A wide range of amino acids, represented by glutamate, leucine, and isoleucine, were highly positively correlated with each other, and soluble proteins also showed consistent positive correlations with these amino acids. This strong positive correlation may be because these amino acids share common metabolic precursors and are simultaneously required for protein synthesis [39,40]. In contrast, soluble proteins and amino acids tended to be negatively correlated with soluble sugars and polysaccharides. This negative correlation may be because soluble proteins and amino acids compete with soluble sugars and polysaccharides for photosynthetic assimilates [41]. The uniform growing conditions largely excluded environmental heterogeneity. Therefore, the higher levels of nitrogenous primary metabolites and vitamins C/E in SY may reflect the role of genetic factors. Overall, SY shows potential for functional food development and would serve as a valuable resource for improving comprehensive fruit quality in breeding programs.

LC ranked second in both PCA and EW-GRA evaluations. It showed higher levels of total phenolics, polysaccharides, and antioxidant activity. These traits indicate an advantage in antioxidant-related functional components. Antioxidant capacity is an important functional indicator of fruit quality [42,43]. Phenolic compounds, especially flavonoids, are widely recognized as major contributors to antioxidant activity due to their strong redox properties and free radical scavenging ability [44,45]. In addition to their antioxidant roles, flavonoids and polysaccharides have also been reported to exert multiple health-promoting effects in humans [46]. Flavonoids are associated with potential benefits such as anti-inflammatory, neuroprotective, and metabolic regulatory activities, which are largely attributed to their ability to modulate oxidative stress and cellular signaling pathways [47]. Similarly, polysaccharides are recognized as major bioactive components in *Lycium* fruits and are involved in immune regulation, anti-inflammatory responses, and the regulation of metabolic-related processes [46]. Under the same cultivation environment, LC exhibited higher total phenolics, polysaccharides, and antioxidant activity. Correlation analysis showed that these indicators were positively associated with fruit size and tended to be negatively correlated with soluble proteins and amino acids. Larger-fruited accessions (including LC) tended to accumulate more carbohydrates and antioxidant activity. Therefore, the higher levels of total phenolics, polysaccharides, and antioxidant activity in LC may be associated with genetic factors. Thus, LC shows potential for the development of functional germplasm resources, particularly in relation to antioxidant-related traits, and may serve as a valuable material for improving fruit functional quality in breeding programs.

## 4. Materials and Methods

### 4.1. Plant Materials and Fruits Collection

The plants from eight natural germplasm resources of *L. chinense* were obtained from its main natural range in the eastern coastal region of China, including Liaocheng city (LC), Rugao city (RG), Dafeng district (DF), Suzhou city (SZ), Qidong city (QD), Dongtai city (DT), Jingjiang city (JJ), and Sheyang county (SY). These plants were transplanted at Yancheng Forest Farm (33.58° N, 120.47° E, Yancheng City) (Figure S1). All plants were wild adult individuals, excavated from their original habitats and directly transplanted to the Yancheng Forestry Station. In the first year after transplantation, the plants grew well, produced abundant fruits, and were fully acclimatized to the local environment. Fruit quality evaluation was then carried out in the second year. According to the description of fruit ripening of *L. chinense* [48], the fruits reach the fully ripe stage (Figure 1). At this fully ripe stage, only red ripe fruits that were uniform in size, bright in color, free from insect damage, and in good growth condition were randomly hand harvested on a single day. For each group, 150 mature fruits were harvested from six plants. For each biological replicate, two plants were used, resulting in three biological replicates in total. For each replicate, the 50 fruits collected from the two plants were pooled and mixed evenly to form a pooled sample. For each replicate, ten fruits were used for immediate measurements of morphological traits. Fruit length and width were measured using a digital vernier caliper (DL3944ABC, Deli, Ningbo, China), and fruit fresh weight was determined using an analytical balance (MA104E, Mettler Toledo, Greifensee, Switzerland). All morphological measurements were expressed as mean values of the three biological replicates. The remaining fruits from each biological replicate were wrapped in aluminum foil and immediately frozen in liquid nitrogen, then subsequently transferred to −80 °C for storage. The frozen samples were ground into fine powder in liquid nitrogen using a ball mill (GT300, Beijing Grinder Instrument Co., Ltd., Beijing, China) and stored at −80 °C for subsequent analyses. Approximately 100 mg of frozen powder from each sample was weighed and dried to determine the fresh-to-dry mass ratio. The remaining frozen powder was used for the determination of other indicators, including amino acids, vitamin C, vitamin E, total phenolics, flavonoids, polysaccharides, and antioxidant activity.

### 4.2. Analysis of Total Phenolics

Total phenolics were determined according to the method described previously with minor modifications and expressed as mg gallic acid equivalents per gram of dry weight (mg GAE g^−1^ DW) [49]. Briefly, fresh samples were extracted with 70% ethanol (Xilong Scientific, Guangzhou, China), and ultrasonicated for 90 min using an ultrasonic cleaner (Kun Shan Ultrasonic Instruments, Kunshan, China). After using a centrifuge (Eppendorf AG, Hamburg, Germany) at 9000 rpm 4 °C for 5 min, an aliquot (0.1 mL) of the supernatant was diluted ten-fold and mixed with 6.0 mL of deionized water and 1.0 mL of Folin–Ciocalteu reagent 1.0 mol L^−1^ (Feijing Biological, Fuzhou, China). 6 min later, 4.0 mL of 10.6% sodium carbonate (Sinopharm Chemical Reagent, Shanghai, China) solution was added. The mixture was incubated in the dark at room temperature for 60 min. The absorbance at 760 nm was determined using a UV-1800 spectrophotometer (Shanghai Mapada Instruments, Shanghai, China).

### 4.3. Analysis of Flavonoids, Polysaccharides and Antioxidant Activity

Flavonoids were determined using a commercial assay kit (Suzhou Comin Biotechnology Co., Ltd., Suzhou, China) according to the manufacturer’s instructions and expressed as mg rutin equivalents per gram of dry weight (mg RE g^−1^ DW). Polysaccharide content and in vitro antioxidant activity were measured using commercial assay kits (Nanjing Kemosen Biotechnology Co., Nanjing, China) according to the manufacturers’ instructions and expressed as mg g^−1^ dry weight (mg g^−1^ DW).

### 4.4. Analysis of Soluble Sugar, Soluble Protein and Carotenoid

Soluble sugar content was determined using a previously reported anthrone colorimetric method with minor modifications [50,51]. Fresh fruit powders were extracted with 80% ethanol and centrifuged (6000× *g*, 10 min). The supernatant was mixed with 0.1% anthrone reagent (Sinopharm Chemical Reagent, Shanghai, China). The mixture was heated in a boiling water bath for 7 min and then cooled to room temperature. Absorbance was measured at 620 nm.

Soluble protein content was determined using a commercial assay kit (Nanjing Kemosen Biotechnology Co.) according to the manufacturer’s instructions.

Carotenoid content was measured using a commercial carotenoid assay kit (Nanjing Kemosen Biotechnology Co.) following the manufacturer’s instructions. All results were expressed as mg g^−1^ dry weight (mg g^−1^ DW).

### 4.5. Analysis of Vitamin C and Vitamin E

Vitamin C content was determined using a previously reported molybdenum blue colorimetric method with minor modifications [52]. Frozen samples were homogenized with 0.05 mol L^−1^ oxalic acid–0.2 mmol L^−1^ EDTA solution (Sinopharm Chemical Reagent, Shanghai, China). The homogenate was extracted by ultrasonication for 30 min and centrifuged at 4000 rpm for 15 min. An aliquot (1.0 mL) of the supernatant was reacted with a mixture of metaphosphoric acid (Macklin Biochemical, Shanghai, China), acetic acid, sulfuric acid, and ammonium molybdate (Sinopharm Chemical Reagent, Shanghai, China). The reaction mixture was incubated at 30 °C for 15 min, and the absorbance was measured at 760 nm.

Vitamin E content was measured using a commercial vitamin E assay kit (Nanjing Kemosen Biotechnology Co.) according to the manufacturer’s instructions. All results were expressed as mg g^−1^ dry weight (mg g^−1^ DW).

### 4.6. Analysis of Total Acidity

Total acidity was determined using an acid–base titration method with minor modifications [53]. The frozen samples were extracted with CO_2_-free water and an aliquot was titrated with 0.1 mol L^−1^ NaOH (Sinopharm Chemical Reagent, Shanghai, China). Total acidity was calculated based on the volume of NaOH consumed and expressed as citric acid equivalents using a conversion factor of 0.070. All results were expressed as mg citric acid equivalents per gram of dry weight (mg CAE g^−1^ DW).

### 4.7. Analysis of Amino Acid

The concentrations of amino acids were determined using Waters Alliance e2695 HPLC system (Waters, Milford, MA, USA) according to previously reported methods [54,55]. The frozen samples were hydrolyzed with 6 mol L^−1^ hydrochloric acid (Sinopharm Chemical Reagent, Shanghai, China) at 110 °C. The hydrolysates were neutralized, filtered, and analyzed by HPLC under standard operating conditions. Amino acid concentrations were quantified using an external standard method. All amino acid concentrations were expressed as mg g^−1^ dry weight (mg g^−1^ DW).

### 4.8. PCA, EW–GRA

PCA was performed to identify key evaluation indicators [56]. Comprehensive scores and sample rankings were calculated based on the variance contribution rates of each principal component using Formula (1), where $F_{i}$ represents the score of the i-th principal component (or factor), and $W_{i}$ denotes the corresponding normalized weight. The PCA results were visualized using the ggplot2 package in R (http://www.r-project.org/ (accessed on 15 February 2026)).

(1) $$F=\sum_{i=1}^{5}W_{i}F_{i}$$

For comprehensive evaluation, the EW method was applied to determine the objective weights of each indicator based on the variability of the standardized data. GRAwas used to evaluate different germplasm resources.

### 4.9. Statistical Analysis

Statistical analysis was performed according to previously reported methods [57]. Data normality was verified prior to analysis. One-way analysis of variance (ANOVA) was conducted using Statgraphics (STN, St. Louis, MO, USA), and differences between means were considered significant at *p* < 0.05 based on the ANOVA F-test. Correlation analysis among all phenotypic and physiological indicators was performed based on previously reported methods with minor modifications [58], using R (http://www.r-project.org/ (version 4.5.2)) and the pheatmap package to examine relationships between variables.

## Figures and Tables

**Table 1:** Composition matrix in the fruit of eight *L. chinense* germplasms.

	Principal Component				
Trait	1	2	3	4	5
Biomass of individual fruit	−0.261	0.702	0.391	0.109	0.428
Length of individual fruit	−0.051	0.157	0.475	−0.170	0.782
Width of individual fruit	−0.464	0.810	0.134	0.230	−0.132
Total phenolics	0.318	−0.420	0.302	0.559	0.466
Flavonoids	0.107	−0.366	0.712	−0.262	−0.331
Polysaccharides	−0.212	0.330	−0.215	0.749	0.223
TEAC	0.473	0.690	0.373	0.191	0.146
Soluble sugar	−0.310	−0.395	−0.202	−0.190	0.387
Soluble protein	0.618	−0.334	0.017	−0.076	0.414
Carotenoid	−0.086	−0.443	0.388	−0.087	0.481
Vitamin C	0.290	−0.266	−0.383	0.559	0.101
Vitamin E	0.169	−0.607	0.225	0.438	−0.356
Total acidity	0.673	−0.260	0.356	−0.385	0.283
Aspartic acid	0.868	−0.040	0.047	0.425	−0.118
Glutamic acid	0.887	0.220	0.183	−0.144	−0.301
Arginine	0.926	0.006	0.206	0.061	−0.270
Alanine	0.876	0.291	0.139	0.236	0.018
Tyrosine	0.899	−0.229	−0.045	0.000	0.217
Glycine	0.963	0.183	−0.001	0.028	−0.101
Serine	0.549	−0.100	−0.420	0.245	0.357
Proline	0.249	0.524	−0.211	−0.626	0.166
Threonine	0.977	0.052	−0.136	−0.058	−0.043
Leucine	0.952	0.095	0.082	−0.023	−0.223
Lysine	0.895	−0.050	−0.272	−0.211	0.211
Phenylalanine	0.960	−0.064	0.056	−0.188	−0.118
Isoleucine	0.935	0.068	0.135	0.078	−0.257
Histidine	0.830	0.215	−0.382	0.144	0.273
Valine	0.846	0.159	−0.283	−0.268	0.189
Methionine	0.086	0.064	0.838	0.253	0.056
The eigenvalue	12.854	3.645	3.064	2.735	2.658
Rate of contribution (%)	44.323	12.568	10.564	9.430	9.165
The cumulative contribution rate (%)	44.323	56.891	67.455	76.885	86.049

**Table 2:** Principal component score and comprehensive score in the fruits of eight *L. chinense* germplasms.

Germplasm	Principal Component 1 Score	Principal Component 2 Score	Principal Component 3 Score	Principal Component 4 Score	Principal Component 5 Score	Comprehensive Scores	Ranking
SY	0.506	0.051	0.054	0.009	0.027	0.647	1
LC	0.182	0.166	0.050	0.016	0.032	0.445	2
RG	0.245	0.081	−0.001	0.020	0.027	0.371	3
QD	0.284	0.073	−0.050	0.012	0.028	0.346	4
DT	0.108	0.041	0.031	0.045	0.087	0.312	5
SZ	0.171	0.082	0.027	−0.063	0.054	0.271	6
DF	0.088	0.055	0.023	0.045	0.004	0.215	7
JJ	0.007	0.046	0.030	−0.014	0.001	0.071	8

**Table 3:** Entropy weight method to determine the weight of 29 evaluation indicators.

Trait	Entropy Value	Coefficient of Variance	Weights
Biomass of individual fruit	0.945	0.055	0.024
Length of individual fruit	0.892	0.108	0.047
Width of individual fruit	0.964	0.036	0.015
Total phenolics	0.926	0.074	0.032
Flavonoids	0.836	0.164	0.071
Polysaccharides	0.950	0.050	0.022
TEAC	0.932	0.068	0.030
Soluble sugar	0.960	0.040	0.017
Soluble Protein	0.944	0.056	0.024
Carotenoid	0.955	0.045	0.020
Vitamin C	0.911	0.089	0.039
Vitamin E	0.959	0.041	0.018
Total acidity	0.942	0.058	0.025
Aspartic acid	0.918	0.082	0.035
Glutamic acid	0.886	0.114	0.050
Arginine	0.913	0.087	0.038
Alanine	0.909	0.091	0.039
Tyrosine	0.944	0.056	0.024
Glycine	0.921	0.079	0.034
Serine	0.941	0.059	0.025
Proline	0.880	0.120	0.052
Threonine	0.929	0.071	0.031
Leucine	0.916	0.084	0.036
Lysine	0.921	0.079	0.034
Phenylalanine	0.933	0.067	0.029
Isoleucine	0.890	0.110	0.048
Histidine	0.938	0.062	0.027
Valine	0.929	0.071	0.031
Methionine	0.812	0.188	0.082

**Table 4:** Weighted correlation of fruit quality and ranking of eight *L. chinense* germplasms.

Germplasms	Weighted Relevance	Ranking
SY	0.712	1
LC	0.532	2
QD	0.511	3
SZ	0.497	4
RG	0.465	5
DT	0.456	6
DF	0.414	7
JJ	0.408	8

## Data Availability

The original contributions presented in this study are included in the article/supplementary material. Further inquiries can be directed to the corresponding authors.

## Supplementary Materials

The following supporting information can be downloaded at: https://www.mdpi.com/article/10.3390/plants15101506/s1, Figure S1: Geographical information of collection sites for eight germplasms of wild *L. chinense*.
