# Supplementary material for: Evaluation of Lycium chinense Germplasms in China Based on Fruit Quality Traits
Source: Plants (Basel). 2026 May 15;15(10):1506. doi: 10.3390/plants15101506 (PMC13211219; doi:10.3390/plants15101506)
Supplement: Supplementary file 1 [file plants-15-01506-s001.zip › plants-4292438-supplementary.pdf]

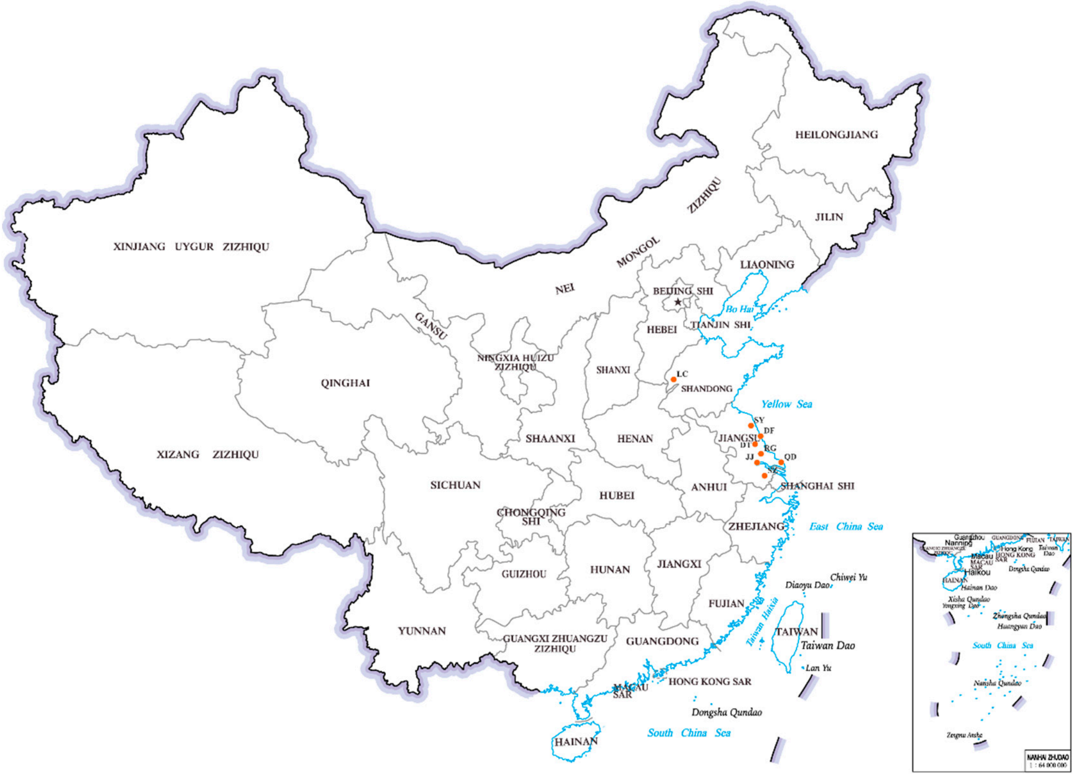

Figure S1. Geographical information of collection sites for eight germplasms of wild *L. chinense*. Red dots represent the collection sites.
